# Loss and Natural Variations of Blast Fungal Avirulence Genes Breakdown Rice Resistance Genes in the Sichuan Basin of China

**DOI:** 10.3389/fpls.2022.788876

**Published:** 2022-04-12

**Authors:** Zi-Jin Hu, Yan-Yan Huang, Xiao-Yu Lin, Hui Feng, Shi-Xin Zhou, Ying Xie, Xin-Xian Liu, Chen Liu, Ru-Meng Zhao, Wen-Sheng Zhao, Chuan-Hong Feng, Mei Pu, Yun-Peng Ji, Xiao-Hong Hu, Guo-Bang Li, Jing-Hao Zhao, Zhi-Xue Zhao, He Wang, Ji-Wei Zhang, Jing Fan, Yan Li, Yun-Liang Peng, Min He, De-Qiang Li, Fu Huang, You-Liang Peng, Wen-Ming Wang

**Affiliations:** ^1^State Key Laboratory of Crop Gene Exploration and Utilization in Southwest China, Sichuan Agricultural University, Chengdu, China; ^2^State Key Laboratory of Agrobiotechnology and Ministry of Agriculture Key Laboratory of Plant Pathology, College of Plant Protection, China Agricultural University, Beijing, China; ^3^Plant Protection Station, Department of Agriculture Sichuan Province, Chengdu, China; ^4^Institute of Plant Protection, Sichuan Academy of Agricultural Sciences, Chengdu, China

**Keywords:** rice blast, *Magnaporthe oryzae*, avirulence gene, variation, monogenetic lines, resistance gene

## Abstract

*Magnaporthe oryzae* is the causative agent of rice blast, a devastating disease in rice worldwide. Based on the gene-for-gene paradigm, resistance (R) proteins can recognize their cognate avirulence (AVR) effectors to activate effector-triggered immunity. *AVR* genes have been demonstrated to evolve rapidly, leading to breakdown of the cognate resistance genes. Therefore, understanding the variation of *AVR* genes is essential to the deployment of resistant cultivars harboring the cognate *R* genes. In this study, we analyzed the nucleotide sequence polymorphisms of eight known *AVR* genes, namely, *AVR-Pita1, AVR-Pii, AVR-Pia, AVR-Pik, AVR-Pizt, AVR-Pi9, AVR-Pib*, and *AVR-Pi54* in a total of 383 isolates from 13 prefectures in the Sichuan Basin. We detected the presence of *AVR-Pik, AVR-Pi54, AVR-Pizt, AVR-Pi9*, and *AVR-Pib* in the isolates of all the prefectures, but not *AVR-Pita1, AVR-Pii*, and *AVR-Pia* in at least seven prefectures, indicating loss of the three *AVR*s. We also detected insertions of Pot3, Mg-SINE, and indels in *AVR-Pib*, solo-LTR of Inago2 in *AVR-Pizt*, and gene duplications in *AVR-Pik*. Consistently, the isolates that did not harboring *AVR-Pia* were virulent to IRBLa-A, the monogenic line containing *Pia*, and the isolates with variants of *AVR-Pib* and *AVR-Pizt* were virulent to IRBLb-B and IRBLzt-t, the monogenic lines harboring *Pib* and *Piz-t*, respectively, indicating breakdown of resistance by the loss and variations of the avirulence genes. Therefore, the use of blast resistance genes should be alarmed by the loss and nature variations of avirulence genes in the blast fungal population in the Sichuan Basin.

## Introduction

Rice blast disease is one of the most destructive fungal diseases in rice worldwide caused by the filamentous fungal pathogen *Magnaporthe oryzae* (*M. oryzae*) (syn. *Pyricularia grisea* B. C. Couch) (Talbot, 2003; Dean et al., 2012). Rice blast disease causes 10 to 35% yield loss each year, even no yield in some regions with severe epidemics (Dean et al., 2012; Fisher et al., 2012). Developing and application of resistant cultivars are the most effective and environmental-friendly method to control the disease. To date, more than 100 blast resistance (*R*) genes have been mapped and 38 of them have been cloned (Wang et al., 2017; Zhao et al., 2018; Xie et al., 2019; Liu et al., 2020). According to the gene-for-gene paradigm, cultivars that harbor major blast *R* genes can prevent the infection of strains carrying the corresponding avirulence (*AVR*) genes, which are established on the recognitions between *R* genes and *AVR* genes (Silue et al., 1992). However, *AVR* genes are highly variable and the variated AVR protein escape from the recognition mediated by cognate R protein, resulting in breakdown of the resistance function of the *R* gene (Deng et al., 2017). The blast population can overcome the *R* gene-mediated resistance in the field due to the heavy selection pressure caused by the monocropping of a single-resistant cultivar for years (Skamnioti and Gurr, 2009; Valent and Khang, 2010). Accordingly, analyzing the variations of *AVR* genes can evaluate the efficacy of cognate resistance genes (Selisana et al., 2017). Therefore, it is an effective measure to predict *R* genes efficacy and prevent blast pandemics by monitoring the variation of *AVR* genes in the field blast fungal population.

To date, more than 40 *AVR* genes have been identified in the blast fungus and 12 of them (*PWL1, PWL2, ACE1, AVR1-CO39, AVR-Pita1, AVR-Pii, AVR-Pia, AVR-Pik, AVR-Pizt, AVR-Pi9, AVR-Pib*, and *AVR-Pi54*) have been cloned (Kang et al., 1995; Sweigard et al., 1995; Farman and Leong, 1998; Orbach et al., 2000; Fudal et al., 2005; Ma et al., 2006; Li et al., 2009; Yoshida et al., 2009; Wu et al., 2015; Zhang et al., 2015; Ray et al., 2016). The avirulent function of *AVR* genes can be invalidated by different mechanisms, including mutation, genetic recombination, and sexual mating (Noguchi et al., 2006; Tsujimoto Noguchi, 2011). Mutation commonly occurs, such as insertion, point mutation, and deletion. Transposable elements (TEs) can enrich the population diversity through deleting/inactivating genes or horizontal gene transfer (Chuma et al., 2011; Yoshida et al., 2016). Transposon insertion often happens in the promoter or genomic region. For example, Pot3 has been identified as a major reason to generate different variations of *AVR-Pita1, AVR-Pib, AVR-Pii, AVR-Pizt*, and *AVR-Pia* (Kang et al., 2001; Yasuda et al., 2006; Li et al., 2009; Yoshida et al., 2009; Singh et al., 2014; Olukayode et al., 2019). Point mutation of *AVR-Pita1* and *AVR-Pik* could generate novel alleles and some of the alleles can escape from the recognition of the cognate *R* gene (Yoshida et al., 2009; Dai et al., 2010; Kanzaki et al., 2012; Longya et al., 2019; Damchuay et al., 2020). Segmental deletion of coding sequence also results in virulent mutation of *AVR-Pita1* and *AVR-Pib* (Orbach et al., 2000; Zhang et al., 2015). The complete deletion of *AVR* genes was found in *AVR-Pita1, AVR-Pii*, and *AVR-Pia*, generating virulence strains to cultivars harboring their cognate *R* genes (Yoshida et al., 2009). *AVR-Pita1* and *AVR-Pii* locate adjacently to the telomere of their chromosomes; therefore, loss of chromosome tips is the reason for their frequent spontaneous loss (Yasuda et al., 2006; Khang et al., 2008; Li et al., 2009). Moreover, homologous recombination can cause DNA rearrangement, deletion, translocation, and even horizontal gene transfer between strains. For example, homologous recombination between two repetitive sequences results in the deletion of *AVR-Pia* (Sone et al., 2013). Parasexual recombination and sexual mating can also result in the genetic exchange of DNA and they are reasons for the pathogenicity variation of rice blast fungus. Therefore, dynamic adaptation and evolution of rice blast fungus could be achieved through these variations of *AVR* genes in a population, leading to the breakdown of *R* genes.

The Sichuan Basin is one major base of rice production in southwestern China. There are more than 1.5 million hm^2^ paddy fields and the production is enough for feeding 100 million people (Wang and Valent, 2009). The climate of the basin is warm and humid, which is greatly conducive to the epidemics of rice blast disease. In the past several decades, three serious rice blast epidemics have been happened with around 10 years interval, since the first epidemic happened in 1984 and 1985, caused by long-term large-scale monocropping of a single-resistant cultivar Shanyou-2 (Lu et al., 2017). Therefore, surveillance of the loss and variations of the *AVR* genes in the blast fungal population is vital to guarantee rice production in this area.

In this study, we analyzed the distribution and the variation of eight *AVR* genes, namely, *AVR-Pi9, AVR-Pi54, AVR-Pita1, AVR-Pib, AVR-Pia, AVR-Pizt, AVR-Pii*, and *AVR-Pik*, in 383 *M. oryzae* isolates from 13 prefectures in the Sichuan Basin. We tested the pathogenicity of strains that did not contain an *AVR-Pia* gene or contain the *AVR-Pib* and *AVR-Pizt* variants to the monogenic lines harboring their cognate *R* genes. Our data indicate that both the loss and variation of an *AVR* gene in a strain led to breakdown of its cognate *R* gene. Therefore, the use of *Pia, Pib*, and *Piz-t* should be highly alarmed in the Sichuan Basin.

## Materials and Methods

### Fungal Isolates and Culture

**Figure 1:**
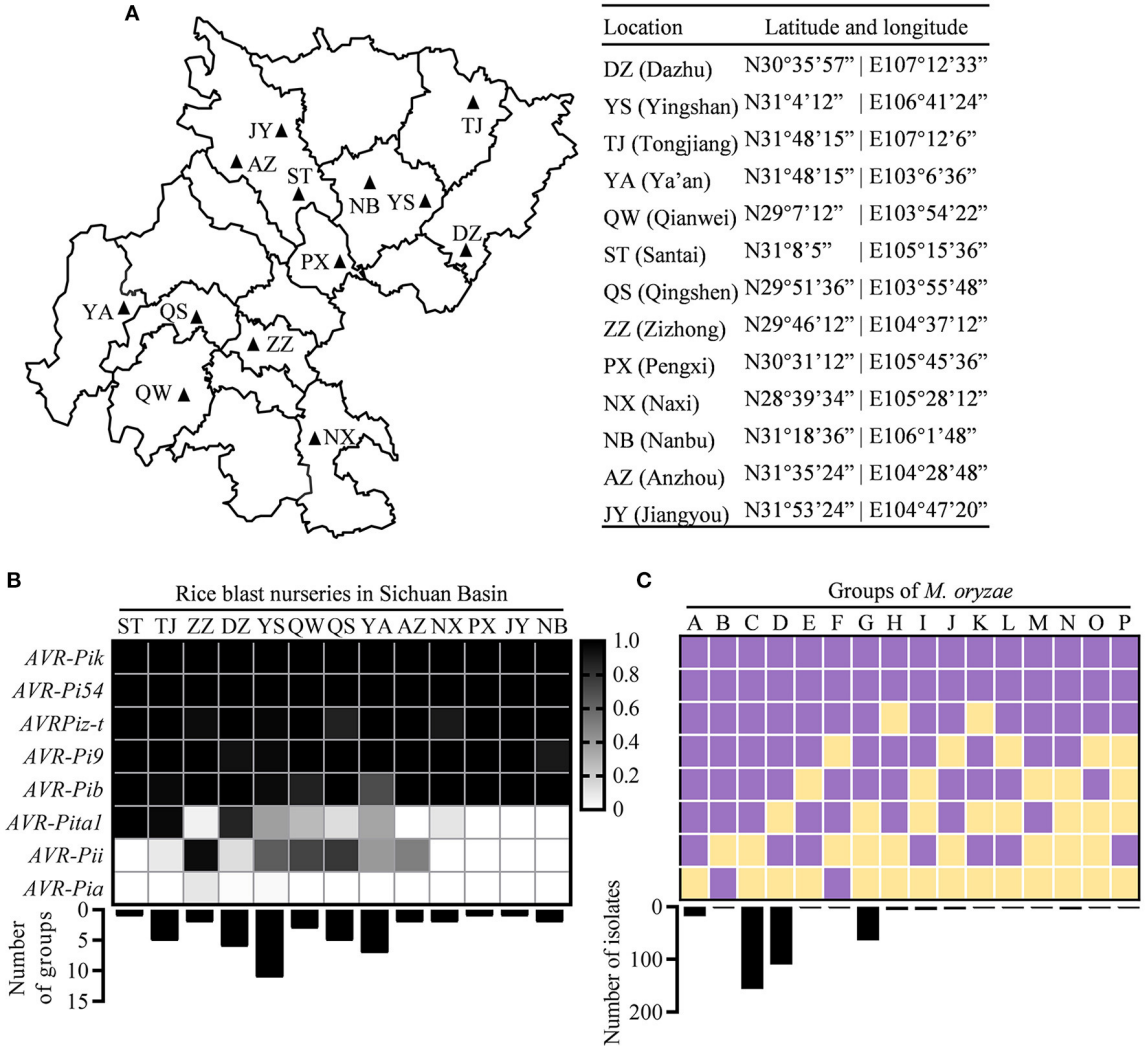
Distribution of avirulence (*AVR*) genes in the Sichuan Basin. **(A)** Location of rice blast nurseries in the Sichuan Basin. **(B)** The distribution of eight *AVR* genes in the indicated nurseries. The presence of each *AVR* gene was indicated by shaded box (shadow key) in the 383 field isolates from 13 prefectures (top) where the strains formed different number of groups (bottom). **(C)** The classification of *Magnaporthe oryzae* (*M. oryzae*) strains based on the presence of *AVR* genes. Upper panel shows the presence/absence of each *AVR* gene and lower panel shows the number of isolates in each group. Purple, presence; yellow, absence.

We collected rice blast-infected leaf samples from rice blast hotspots in 13 nurseries of the Sichuan Basin, including DZ, YS, TJ, YA, QW, ST, QS, ZZ, PX, NX, NB, AZ, and JY (Figure 1A). Then, single-spore isolation method was used to establish single-spore isolates following a previous report (Jia, 2009). A total of 383 single-spore isolates were obtained from the infected leaves of the susceptible variety Lijiangxin Tuan Heigu (LTH). Isolates were cultured on PDA medium (200 g/l potato, 20 g/l dextrose, and 13 g/l agar) in a growth chamber at 25°C in a 12-h light/12-h dark photoperiod for 7 days and were stored at −20°C on filter paper.

### Deoxyribonucleic Acid Preparation, PCR Amplification, and Sequencing

The fungal isolates were cultured on PDA medium in plates covered with cellophane membrane at 25°C in a 12-h light/12-h dark photoperiod for 7 days. Then, mycelia were harvested and frozen in liquid nitrogen for grinding to fine powder. Total genomic DNA was extracted *via* cetyltrimethylammonium bromide (CTAB) method [10 mM Tris-HCl, pH 8.0, 1 mM ethylenediaminetetraacetic acid (EDTA), 100 mM NaCl, 2% sodium dodecyl sulfate (SDS)] for all the fungal isolates (Saghai-Maroof et al., 1984). PCR was performed with gene-specific primers (Supplementary Table 1) for amplifying eight avirulence genes, namely, *AVR-Pib, AVR-Pik, AVR-Pia, AVR-Pi9, AVR-Pi54, AVR-Pita1, AVR-Pizt*, and *AVR-Pii* from isolated DNA samples. Each PCR reaction contains 2.5 μl of 10X *EasyTaq* buffer, 0.25 μl of 5 U/μl *EasyTaq* DNA polymerase (TransGen Biotech Corporation Ltd., Beijing, China), 0.5 μl of 10 mM deoxynucleotide triphosphates (dNTPs), 0.5 μl of each 10 μM primer, 1 μl of 20–50 ng/μl fungal genomic DNA, and 19.5 μl of distilled water. Amplifications were performed in the Bio-Rad Thermal Cycler (C1000, Bio-Rad Laboratories, Life Science Research, California, USA) with the following PCR program: 95°C for 3 min, 30 cycles at 95°C for 30 s, 52 to 58°C for 30 s, 72°C for 30 s, and a final extension at 72°C for 8 min. PCR products were examined by electrophoresis in 1% agarose gel using 120 V for 30 min and the gel was stained with GelRed Nucleic Acid Stain (Biotium, USA). The sizes of PCR amplicons were estimated by Trans2000 DNA Ladder (TransGen Biotech Corporation Ltd., Beijing, China). The PCR amplicons were sequenced by TsingKe Biotech Corporation Ltd. (Beijing, China). The obtained sequences were aligned with the sequences of each *AVR* gene to detect natural variations.

### Plant Growth and Pathogenicity Assay

Rice blast fungal isolates on PDA medium were cultured on OTA media (50 g/l oatmeal, 200 ml/l tomato juice, and 15 g/l agar) at 25°C with 12-h light followed by 12-h dark treatment for producing mycelium. 7 days later, the mycelia were scraped with a cell spreader and the plates were exposed to continuous light at 28°C for 3 to 5 days to promote sporulation. Then, the conidia were washed with 2 ml double-distilled water (ddH_2_O) per petri dish and the suspension was adjusted the concentration to 1 × 10^5^ conidia/ml.

The indicated lines and susceptible control LTH were used for pathogenicity assays in laboratory. The rice seeds were immersed in water for 2 days at 37°C in darkness for germination and then grown in the greenhouse with 28/24 ± 1°C day/night temperature, 70% relative humidity, and 14-h/10-h light/dark period. At three-leaf to five-leaf seedling stage, the second youngest leaves were detached and placed on the ddH_2_O with 6-benzylaminopurinehydrochloride (6-BA) in square petri dish after punch wounded. Then, the punch-wounded leaves were drop inoculated with 10 μl of spore suspension at the wound sites as a previous report (Kong et al., 2012). The inoculated leaves in petri dish were incubated in dark at 26°C in the greenhouse with 90% relative humidity for 24 h (Fang et al., 2018). Lesion size was examined at 4 to 7 days after inoculation to assess the pathogenicity of isolates.

### Data Analysis

The DNA sequences were assembled and aligned with DNAMAN9 (Lynnon Biosoft) and the reference sequences of *AVR* genes were obtained from the National Center for Biotechnology Information (NCBI) with GenBank accessions given in Supplementary Table 1.

## Results

### Presence of Avirulence Genes in Field Isolates From the Sichuan Basin

We isolated the single spores from diseased leaves of susceptible variety LTH in 13 nurseries of the Sichuan Basin (Figure 1A). Finally, 383 isolates were obtained and subjected to investigate the variations of *AVR* genes. We analyzed the nucleotide sequence polymorphisms of eight known *AVR*s, namely, *AVR-Pi9, AVR-Pi54, AVR-Pita1, AVR-Pib, AVR-Pia, AVR-Pizt, AVR-Pii*, and *AVR-Pik*. First, we investigated their presence based on PCR amplification and classified the 383 isolates into the 16 race groups based on the presence of *AVR* genes. We found that five *AVR* genes, namely, *AVR-Pik, AVR-Pi54, AVR-Pizt, AVR-Pi9*, and *AVR-Pib*, were present in all the nurseries and the isolates containing the five *AVR* genes formed the major race groups of *M. oryzae* in the Sichuan Basin (Figures 1B,C). Nevertheless, *AVR-Pita1* was present in the isolates from ST, TJ, and DZ, but almost (the present ratio < 0.1) or completely absent in five rice blast nurseries, including ZZ, AZ, PX, JY, and NB. *AVR-Pii* was present in the isolates of ZZ, but almost or completely absent in six rice blast nurseries, namely, ST, TJ, NX, PX, JY, and NB (Figure 1B). *AVR-Pia* was only present in a few isolates obtained from ZZ, DZ, and YS.

**Figure 2:**
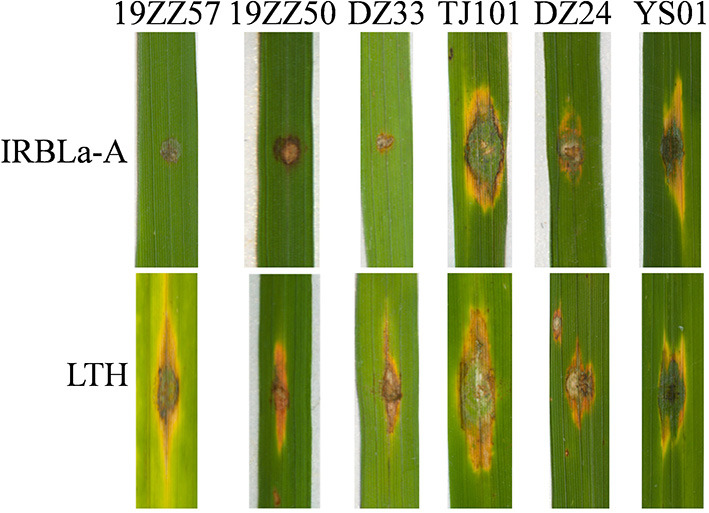
Loss of *AVR-Pia* broke down the resistance of *Pia* in rice. The representative blast disease phenotypes displayed on the leaves of susceptible line Lijiangxin Tuan Heigu (LTH) upon inoculation with the indicated strains. IRBLa-A is the monogenic line harboring the blast resistance gene *Pia*. *AVR-Pia* is present in the three isolates, namely, 19ZZ57, 19ZZ50, and DZ33, but not in the others, TJ101, DZ24, and YS01. The representative figures were taken at 5 days postinoculation (dpi).

We speculated that the isolates that did not contain *AVR-Pia* may become virulent to the monogenic IRBL line carrying cognate *R* gene *Pia*. We inoculated these isolates to IRBLa-A. As our expectation, IRBLa-A showed susceptibility to all the isolates (Figure 2), indicating that loss of *AVR-Pia* breaks down the resistance of *Pia*. Similarly, those strains that did not contain *AVR-Pita1, AVR-Pii*, and *AVR-Pia* may breakdown their cognate *R* genes.

We classified the race group of *M. oryzae* based on their harbored *AVR* genes. The 383 isolates were divided into the 16 race groups (Figure 1C). The groups C and D are the two largest groups consisting of 156 and 110 isolates, respectively. The groups G and A consist of 64 and 18 isolates, respectively. The rest groups each consist of fewer than 10 isolates. To estimate the diversity of *M. oryzae* in each prefecture, we calculated the number of the race groups in each nursery (Figure 1B). There are 11 groups existed in YS, suggesting that it has the most abundant diversity of *M. oryzae*. The rest nurseries have one to seven groups. Among them, the seven groups were identified in YA, while only the one and two groups were identified in ST and ZZ, respectively. There are similar amounts of detected isolates in these nurseries, such as 20 in YA, 19 in ST, and 19 in ZZ (Figure 1A). These data indicate that the diversity of *M. oryzae* shows distinct geographic feature in the Sichuan Basin.

### Sequence Diversity and Distribution of *AVR-Pib*

**Figure 3:**
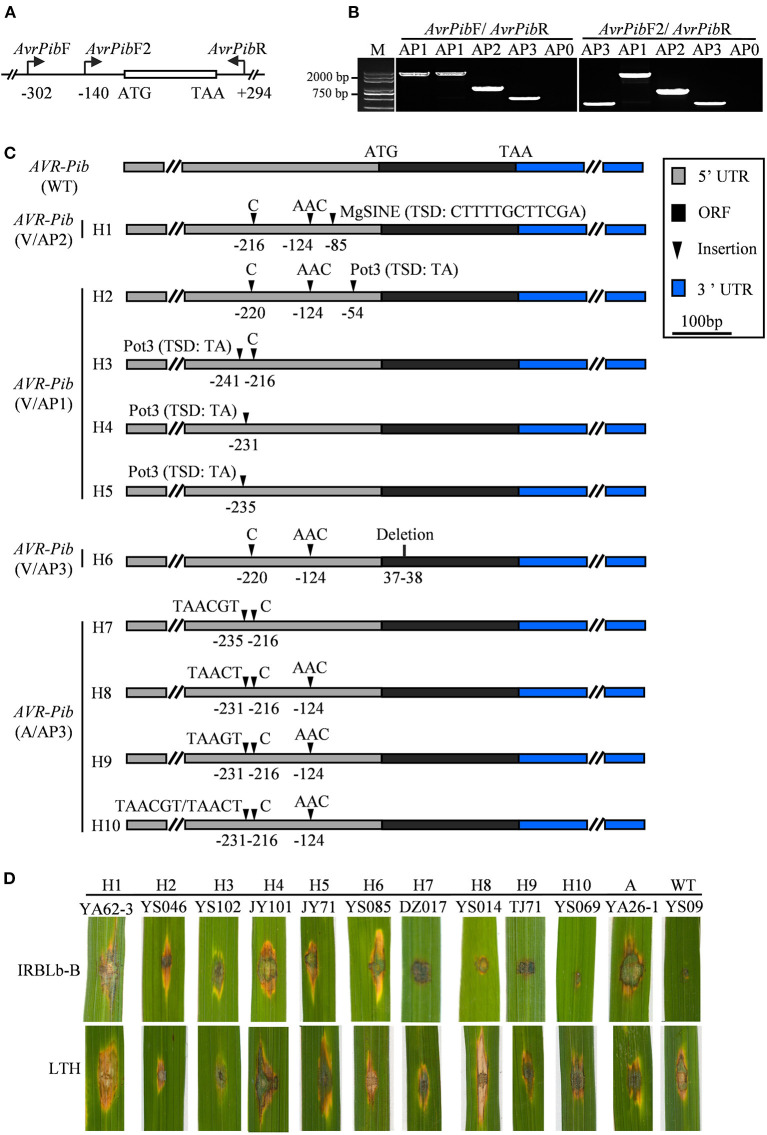
*AVR-Pib* variants and their pathogenicity to IRBLb-B. **(A)** Graph illustrates the location of the primer pairs *AvrPibF*/*AvrPibR* and *AvrPibF2*/*AvrPibR* used to examine the variation in *AVR-Pib* locus. **(B)** Amplification patterns of *AVR-Pib*. Four amplification patterns (AP0-AP3) were classified by the size of the fragments, which amplified from different isolates by indicated primers. AP0 indicates no amplicon. AP1, AP2, and AP3 indicate different sizes of amplicons. M, marker. **(C)** Characterization of allelic variations at *AVR-Pib*. H1 to H10, ten haplotypes of *AVR-Pib* contain different variations. **(D)** Pathogenicity of the indicated isolates on the monogenic line IRBLb-B carrying *Pib*. A, absence of *AVR-Pib* in the indicated strain.

Although *AVR-Pib* has high presence in most isolates (Figure 1B), its cognate *Pib* gene has been lost resistance in the Sichuan Basin (Zhang et al., 2017). Therefore, we investigated the sequence diversity of *AVR-Pib*. The genomic sequence covering the coding sequence (CDS) region was amplified *via* PCR using *AVR-Pib*-specific primers (Figure 3A; Supplementary Table 1). The PCR products can be divided into four different amplification patterns (APs) to denote no amplicon (AP0) and different sizes of amplicons (AP1-PA3) (Figures 3A,B). These APs include 10 variations that are designed haplotype 1 (H1) to haplotype 10 (H10) (Figure 3C). AP1 is the biggest PCR amplicon with over 2,000 bps, which was detected in 235 isolates and is larger than the expected size of *AVR-Pib* (GeneBank: KM887844.1), indicating that there is an insertion. Consistently, sequencing analysis identified an 1,861 bp insertion that shared 99.84% nucleotide sequence identity to the transposon Pot3 (GeneBank: AF333034.1) and flanked with two base pairs (5′-TA-3′) target site duplication (TSD). The insertion locates at four novel sites in 5′-untranslated region (5′-UTR) of *AVR-Pib*, including at −54, −231, −235, and −241 bp upstream of the start codon, which belong to H2, H3, H4, and H5 (Figure 3C). We developed an internal primer pairs, *AvrPib*F2/*AvrPib*, which could distinguish H2 from H3, H4, and H5 (Figures 3A–C). AP2 was detected in seven isolates from YA with near 1,000 bp, which defined as H1. Sequence alignment revealed that AP2 had a 482-bp insertion at the position of −85 bp from the start codon of *AVR-Pib*. This insertion showed 97.1% nucleotide sequence identity to the non-LTR retrotransposon Mg-SINE (GeneBank: U35313.1) and was flanked with a 12-bp sequence (CTTTTGCTTCGA) (Figure 3C). AP3 matches with the expected size of *AVR-Pib* (Figure 3B). Sequencing analysis identified several indels in the CDS and 5′-UTR of *AVR-Pib* (Figure 3C). A deletion occurs at positions 37 and 38 of the CDS region (H6) and several insertions occur in the 5′-UTR, including insertion of AAC at −124, C at −216 or −220, TAACT or TAAGT at −231, and TAACGT at −235 (H7-H9) (Figure 3C). Moreover, the sequencing chromatogram uncovered that some AP3 has double peaks, which decoded into two types of sequences with insertions at −231, −216, and −124 (H10). One sequence has insertion of TAACGT at −231, C at −216, and AAC at −124 and another sequence has insertion of TAACT at −231, C at −216, and AAC at −124 (Figure 3C). Besides, we failed to obtain amplicons from 15 isolates, which were from YS (5 isolates), TJ (2 isolates), YA (6 isolates), and QW (2 isolates) (Supplementary Table 2). These data indicate that the diverse ways were employed by *AVR-Pib* for escaping from R protein detection, such as insertion of Pot3, Mg-SINE, indels, and gene loss.

Then, we tested whether the isolates harboring *AVR-Pib* variations were virulent to IRBLb-B containing cognate *Pi-b*. To this end, we performed pathogenicity assays and found that isolates with short insertions are avirulent to IRBLb-B, including H7, H8, H9, and H10 (Figures 3C,D), indicating that the short sequence insertions in 5′-UTR may not impact the function of *AVR-Pib*. In contrast, the isolates virulent to IRBLb-B carry the other four types of variations, including Mg-SINE insertion (H1), Pot3 insertion (H2-H5), deletion in CDS (H3), and absence of *AVR-Pib* (Figures 3C,D), indicating that these variations caused function loss of *AVR-Pib* to breakdown of the *Pib* resistance.

**Figure 4:**
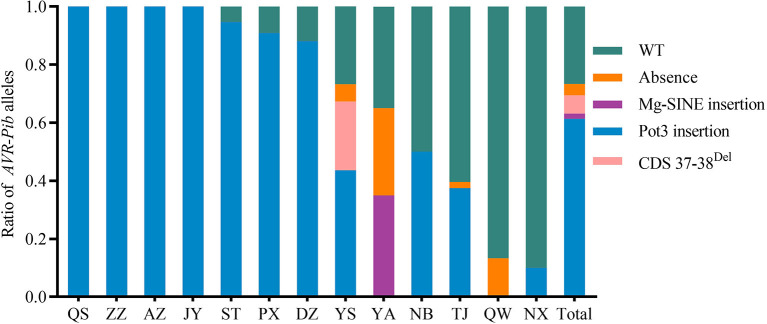
Distribution of isolates carrying variants of invalid *AVR-Pib* in 13 nurseries of the Sichuan Basin. Four variants, namely, absence, Mg-SINE insertion, Pot3 insertion, and coding sequence (CDS) 37-38^Del^, which caused function loss of *AVR-Pib*. Wild type (WT) denotes isolates have similar amplification size of *AVR-Pib* WT. Although some of variants may exist short insertions in the promoter of *AVR-Pib*, it is still avirulence to the monogenic line IRBLb-B carrying *Pib*.

To further investigate the perniciousness of invalid *AVR-Pib* in the Sichuan Basin, we calculated the ratios of *AVR-Pib* variants in all the isolates (Figure 4). Our data indicate that 26.63% isolates contain the valid *AVR-Pib*, i.e., wild type (WT) and short sequence insertions in 5′-UTR of *AVR-Pib*. The invalid *AVR-Pib* exists in 73.37% isolates, consisting with the conclusion that its cognate *Pib* gene has been lost resistance in the Sichuan Basin (Zhang et al., 2017). There are four variations of invalid *AVR-Pib*, Pot3 insertion, 2 bp deletion in CDS (CDS 37–38^Del^), absence, and Mg-SINE insertion, which, respectively, exist in 61.36, 6.27, 3.91, and 1.83% isolates, suggesting that Pot3 insertion is the major way to invalidate *AVR-Pib*. In addition, the isolates carrying Pot3 insertion extensively exist in 11 nurseries, except YA and QW, and preponderantly in seven nurseries, namely, QS, ZZ, AZ, JY, ST, PX, and DZ. Variants with 2 bp deletion in CDS and Mg-SINE insertion of *AVR-Pib* were only detected in part of isolates from YS and YA, respectively. Some isolates lost *AVR-Pib* gene in YS, YA, TJ, and QW. These varieties of invalid *AVR-Pib* were carried by most isolates in YS and YA. However, the valid *AVR-Pib* existed in over half of isolates in QW, NB, NX, and TJ (Figure 4), hinting that the *Pib* gene may still confer resistance in these four regions, especially in QW and NX.

### Variations in *AVR-Pizt* and *AVR-Pita1*

**Figure 5:**
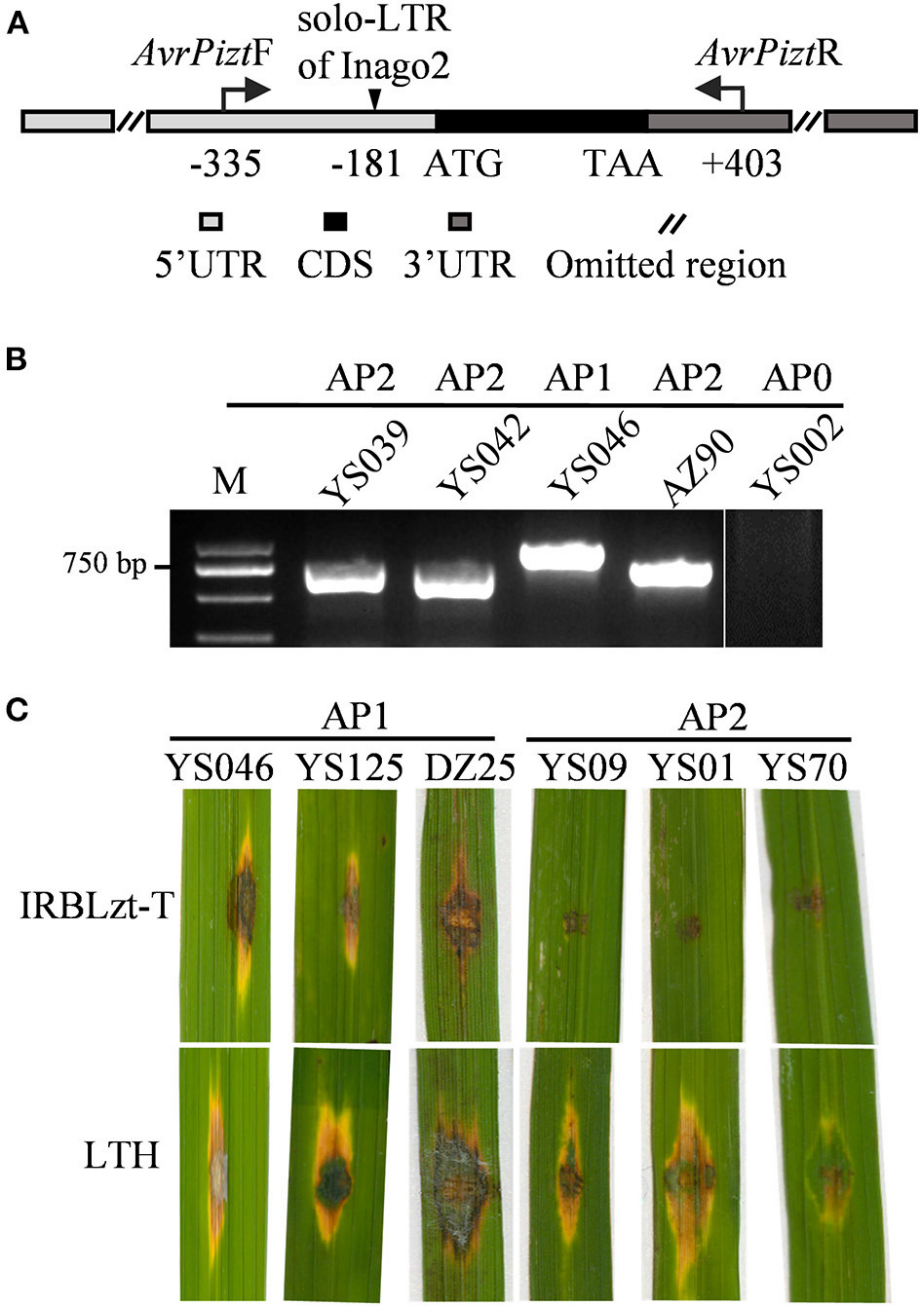
*AVR-Pizt* variants and their pathogenicity to IRBLzt-T. **(A)** Schematic diagram of *AVR-Pizt* and the insert site of a solo-LTR of Inago2. **(B)** Amplification patterns of *AVR-Pizt* from the indicated isolates. The primers *AvrPiztF*/*AvrPiztR* were used to distinguish three alleles. AP0 indicates no amplicon. AP1, AP2, and AP3 indicate different sizes of amplicons. **(C)** Pathogenicity of representative strains on the monogenic line IRBLzt-T carrying *Piz-t*. AP1 strains have an insertion of solo-LTR in *AVR-Pizt* gene and AP2 strains have WT allele of *AVR-Pizt* gene.

Previously, insertions were reported in the 5′-UTR of *AVR-Pizt*, resulting in the function loss of *AVR-Pizt* and breakdown of the *Pizt* resistance (Wang et al., 2020). The fragment was amplified *via* PCR with primers *AvrPiztF* and *AvrPiztR* (Figure 5A). There are two sizes of the amplicons obtained in 97.91% of the isolates, but failed in a few isolates from YS, QS, ZZ, and NX (Figure 5B; Supplementary Table 2). Sequencing analysis indicated that there is an insertion of 201 bp at −181 bp upstream of the start codon of *AVR-Pizt* (Figure 5A). The insertion shared 96% sequence identity to solo-LTR of retrotransposon Inago2 (GeneBank: AB334125.1). The insertion was detected in 59 isolates obtained from the nurseries of YS and DZ (Supplementary Table 2). According to the pathogenicity assays, three representative isolates, namely, YS046, YS125, and DZ25, harboring the insertion are virulent to the monogenic rice blast resistance line IRBLzt-T carrying *Pizt* (Figure 5C), indicating that the insertion disrupts the avirulent function of *AVR-Pizt*, leading to breakdown of the *Pizt*-mediated resistance in the Sichuan Basin.

*AVR-Pita1* locates close to the telomere region on the third chromosome. Some literatures reported that *AVR-Pita1* is easy to be lost or exists as different avirulent haplotypes (Zhou et al., 2007; Singh et al., 2014; Damchuay et al., 2020). In this study, we used gene-specific primers to amplify fragments containing the whole CDS region of *AVR-Pita1* (Supplementary Table 1). The expected size fragment was successfully amplified from 191 (49.9%) isolates. Analyzing the sequence of fragment, we identified five variations for *AVR-Pita1* (Supplementary Table 3). All these variations had been reported to be still avirulent (Li et al., 2014; Damchuay et al., 2020). However, over half of isolates (192, 50.1%) failed to produce an amplificon, indicating that *AVR-Pita1* is losing and going to breakdown the resistance function of *Pita* in the Sichuan Basin.

### Haplotype Identification and Distribution of *AVR-Pik* Alleles

**Figure 6:**
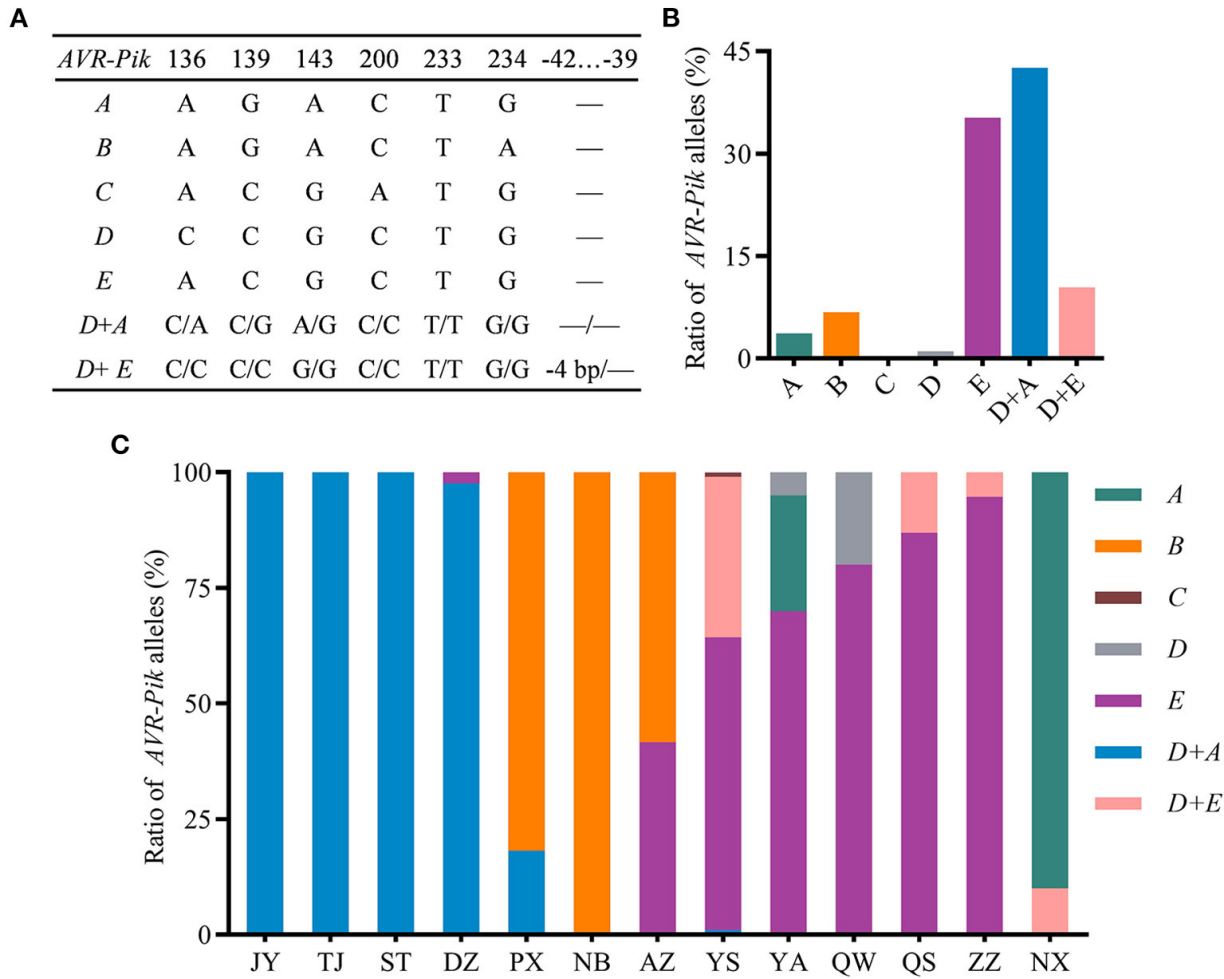
The variation and distribution of *AVR-Pik* alleles in the Sichuan Basin. **(A)** *AVR-Pik* alleles characterized in the isolates from the Sichuan Basin. −4bp, CTTT is deleted at −42 to −39 of the promoter of *AVR-PikD*. —, no change compared with WT. **(B)** Ratio of isolates harboring different *AVR-Pik* alleles. **(C)** Ratio of *AVR-Pik* alleles in the indicated 13 prefectures in the Sichuan Basin.

To investigate the natural variation of *AVR-Pik* in the field isolates of the Sichuan Basin, we analyzed the sequence of CDS region of *AVR-Pik*. The *AVR-Pik* gene was successfully amplified from all the isolates, indicating that *AVR-Pik* exists widely in the isolates from the Sichuan Basin. Sequencing analysis identified five *AVR-Pik* alleles, namely, *AVR-PikA, AVR-PikB, AVR-PikC, AVR-PikD*, and *AVR-PikE* (Figure 6A). Among them, single *AVR-PikA, AVR-PikB, AVR-PikC, or AVR-PikD* was characterized in less than 10% isolates. Especially, *AVR-PikC* is present only in 0.26% isolates. In contrast, *AVR-PikE* is abundant and exists in 35.25% isolates. The rest isolates (53.00%) were characterized as harboring two *AVR-Pik* alleles, namely, *AVR-PikD* + *AVR-PikA* and *AVR-PikD* + *AVR-PikE*, which were detected in 42.56 and 10.44% isolates, respectively (Figure 6B). Therefore, *AVR-PikE* and *AVR-PikD* + *AVR-PikA* are dominant *AVR-Pik* alleles in the blast fungal populations in the Sichuan Basin. In addition, we identified a new deletion of CTTT, which happened at −42 to −39 upstream of the start codon of *AVR-PikD* in the *AVR-PikD* + *AVR-PikE* harbored isolates (Figure 6A).

Meanwhile, we analyzed the distribution of different *AVR-Pik* alleles in the 13 nurseries (Figure 6C). Single *AVR-PikA* was found in isolates from YA (25%) and NX (90%). *AVR-PikB* was detected in isolates from NB (100%), PX (81.82%), and AZ (58.33%). *AVR-PikC* was detected only in one isolate from YS. Single *AVR-PikD* was detected in YA (5%) and QW (20%). Single *AVR-PikE* was detected in five nurseries that accounted for 63.37, 70, 80, 86.96, and 94.74% of the isolates from YS, YA, QW, QS, and ZZ, respectively. Single *AVR-PikE* was also detected in two isolates from DZ and five isolates from AZ. Double alleles of *AVR-PikD* + *AVR-PikA* were detected in DZ (97.62%), YS (0.99%), TJ (100%), ST (100%), PX (18.18%), and JY (100%), whereas another double alleles of *AVR-PikD* + *AVR-PikE* were found in YS (34.65%), QS (13.04%), ZZ (5.26%), and NX (10%). Taken together, *AVR-PikA* was the dominant allele of *AVR-Pik* in the nursery of NX. *AVR-PikB* was dominant in the PX, NB, and AZ. *AVR-PikE* was dominant in the YS, YA, QW, QS, and ZZ. *AVR-PikD* + *AVR-PikA* were dominant in the JY, TJ, ST, and DZ. These data indicate that *AVR-Pik* alleles existed in all the isolates with distinct geographical feature and, thus, cultivars carrying *Pikm* can be exploited in the Sichuan Basin.

### Mating Type of *Magnaporthe oryzae* in the Sichuan Basin

**Figure 7:**
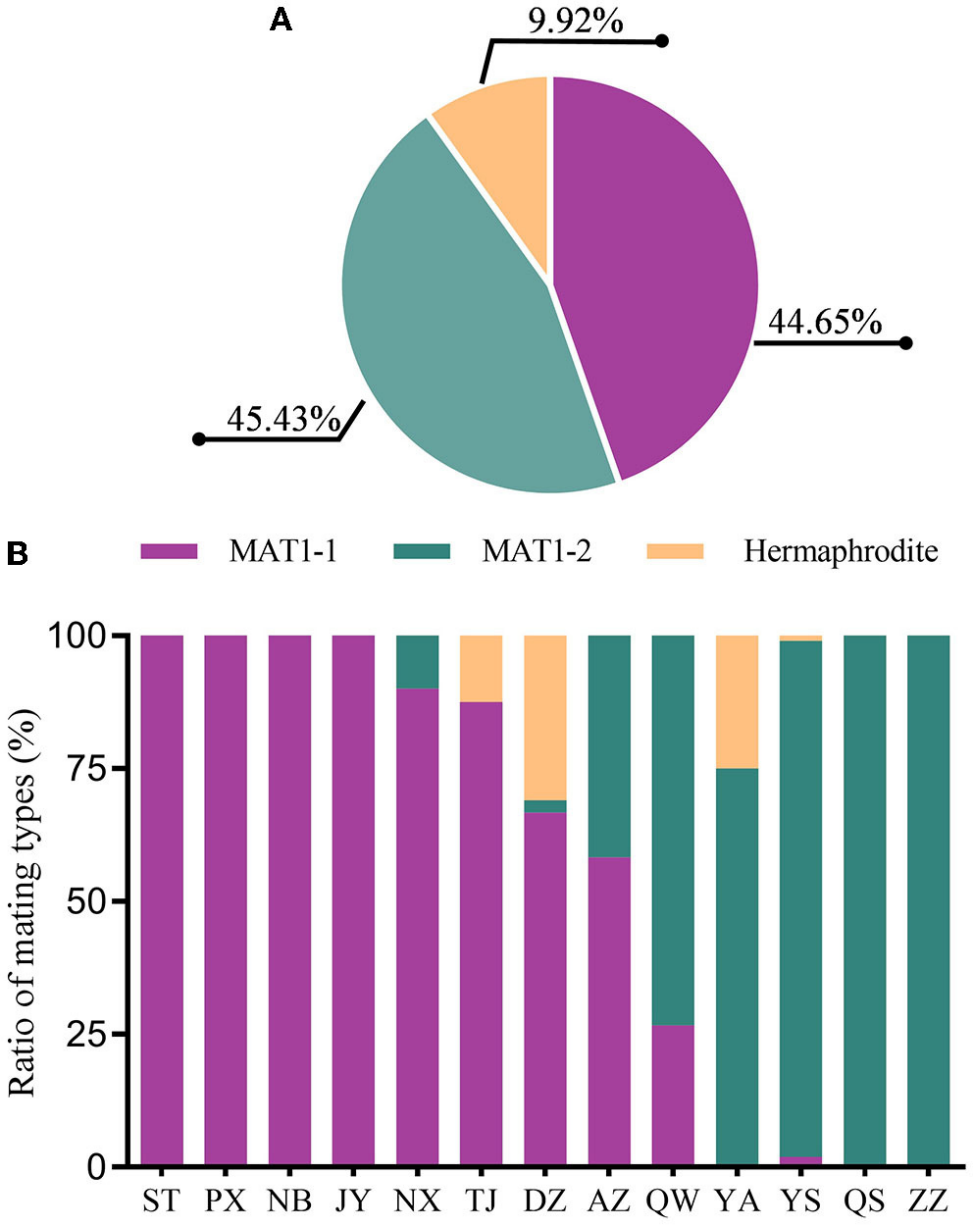
The composition and distribution of MAT1-1 and MAT1-2 isolates in the *M. oryzae* population in the Sichuan Basin. **(A)** The composition of MAT1-1 and MAT1-2 in the isolates. **(B)** The distribution of isolates with different mating types in the Sichuan Basin.

Specific primers for MAT1-1 and MAT1-2 (Supplementary Table 1) were used to investigate the mating type composition of isolates in the *M. oryzae* population. In total, 171 isolates are MAT1-1 and 174 isolates are MAT1-2 (Figure 7A), indicating that the distribution of MAT1-1 and MAT1-2 is relatively balanced in the Sichuan Basin. In addition, we also identified hermaphroditic type in 38 isolates, which have both the mating types simultaneously. Single mating type was found in the isolates from 6 of 13 nurseries, namely, ST, PX, NB, JY, QS, and ZZ. Two mating types were detected in the rest seven nurseries and the hermaphroditic isolates were found in TJ, DZ, and YA (Figure 7B). Even though the sexual status is hardly observed under natural conditions, sexual reproduction potential is still present in regions where the isolates have both the mating types, hinting that the parasexual recombination may be an important way to promote variation of *AVR* genes in the Sichuan Basin.

## Discussion

The dominant population of *M. oryzae* displays distinct geographic feature, which results from the different environments and rice varieties determined by local preferences. As the diversity of rice varieties has abundant, the geographic feature is increasingly apparent in population of *M. oryzae*. Therefore, monitoring the presence and variation of *AVR* genes is an efficient way to crack the geographic code in *M. oryzae* population (Selisana et al., 2017). Usually, commercial cultivars or monogenetic resistance lines were subjected for monitoring. It can select resistance cultivars or clear the valid *R* genes for variety deployment, but cannot reflect the population of *M. oryzae* accurately for prediction of rice blast epidemic. In this study, we collected disease samples from the susceptible rice accession LTH that does not have any known resistance genes, to establish single spore isolates, which are more accurate to represent the populations of *M. oryzae* in paddy fields.

We obtained 383 *M. oryzae* isolates from LTH in 13 nurseries located in the Sichuan Basin. According to the gene-for-gene paradigm, activation of resistance depends on the recognition between R and AVR protein. Loss and variations in *AVR*s can result in breakdown of the cognate *R* genes. To speculate the valid *R* genes in different regions of the Sichuan Basin, we analyzed eight *AVR* genes, namely, *AVR-Pi9, AVR-Pi54, AVR-Pita1, AVR-Pib, AVR-Pia, AVR-Pizt, AVR-Pii*, and *AVR-Pik*, in the 383 isolates. We found that the *AVR* genes of *M. oryzae* employed different mechanisms, such as transposon insertion, short sequence insertion, nucleotide deletion and substitution, gene duplication, as well as gene loss (Figures 1, 3, 5, 6; Supplementary Tables 2, 3), to avoid perception mediated by host *R* genes for pathogenesis in the Sichuan Basin.

The resistance function of *Pib* seems completely invalidated due to the high frequency of loss and sequence variations in *AVR-Pib* in the Sichuan Basin. According to previous reports, IRBLb-B carrying *Pib* is susceptible to more than 60% of *M. oryzae* strains isolated from Cambodia and Hunan province in China (Fukuta et al., 2014; Xing et al., 2017). IRBLb-B also shows susceptible to all the 248 field isolates from Bohol of Philippines because of the prevailing insertion of Pot3 in *AVR-Pib* (Olukayode et al., 2019). *AVR-Pib* seems undergone different genetic events, including transposable elements insertion, segmental deletion, complete absence, and point mutation in 300 isolates collected from the southern, northeastern, and far northeastern regions of China (Zhang et al., 2015). These reports indicated that *AVR-Pib* showed diverse variations in the nature, causing loss of avirulence. In this study, we detected *AVR-Pib* in 96.08% of the isolates from the Sichuan Basin. However, *AVR-Pib* demonstrated abundant variations in different regions of the Sichuan Basin, including effective variations that cause the loss of the avirulent function in *AVR-Pib*. Among these variations, the main variation came from the Pot3 insertions in the 5′-UTR of *AVR-Pib*, which led to breakdown of the *Pib* resistance (Figure 3). Besides, we also identified some novel sites of Pot3 and Mg-SINE insertion, which generated different virulent haplotypes of *AVR-Pib* (Figure 3).

*AVR-Pik* is the most complicated *AVR* gene locus among the eight *AVR*s. To date, six *AVR-Pik* alleles have been identified based on polymorphic amino acids at five sites, namely, *AVR-PikA, AVR-PikB, AVR-PikC, AVR-PikD, AVR-PikE*, and *AVR-PikF* (Yoshida et al., 2009; Longya et al., 2019). Among them, *AVR-PikD* was the most popular allele and it is most likely the ancestral allele, from which the *AVR-PikE, A, C*, and *B* alleles were derived (Yoshida et al., 2009; Kanzaki et al., 2012). *AVR-Pik* alleles can be specifically recognized by different *Pik* genes to trigger immune responses. The ancestral allele *AVR-PikD* can be recognized by five *Pik* alleles (*Pik, Pi-km, Pi-kh, Pi-kp*, and *Pi-ks*). *AVR-PikE* is recognized by three *Pik* alleles (*Pik, Pi-km*, and *Pi-kh*), while *AVR-PikA* is recognized by *Pi-km* and *Pi-kh*. However, all the *Pik* alleles cannot recognize *AVR-PikB, AVR-PikC*, and *AVR-PikF* (Yoshida et al., 2009; Kanzaki et al., 2012; Wu et al., 2014; Zhai et al., 2014; Wang et al., 2017). *AVR-PikD, AVR-PikA, AVR-PikC*, and *AVR-PikE* exist commonly worldwide, but *AVR-PikB* was previously identified only in a Japan isolate and the Chinese isolate Zhong-10-8-14 and *AVR-PikF* is a novel emerging allele reported in Thailand (Yoshida et al., 2009; Kanzaki et al., 2012; Cao et al., 2017; Longya et al., 2019). Here, we did not detect *AVR-PikF* in the Sichuan Basin. We identified five *AVR-Pik* (*A-E*) alleles and found that some isolates harboring two copies of *AVR-Pik* alleles containing *AVR-PikD* + *AVR-PikA* or *AVR-PikD* + *AVR-PikE* (Figure 6; Supplementary Table 2). *AVR-PikD* + *AVR-PikA* were previously reported presumably generated from gene duplication (Wang et al., 2017), *AVR-PikD* + *AVR-PikE* might be a novel gene duplication event occurred to the blast fungal population in the Sichuan Basin. As one of the main fungal adaption and evolution mechanisms, gene duplication can lead to the emergence of novel virulent alleles of *AVR* genes (Chuma et al., 2011). Isolates harboring two copies of *AVR-Pik* are likely to be virulent through losing *AVR-PikD*. Furthermore, virulent alleles *AVR-PikB* and *AVR-PikC* have already occurred in three nurseries (PX, NB, and AZ) and one prefecture (YS) in the Sichuan Basin, respectively (Figure 6C). Isolates harboring different *AVR-Pik* alleles seem gradually become aggressive in the Sichuan Basin.

Parasexual recombination and sexual mating can result in the occurrence of complex rice blast races and arising of pathogenic variants (Noguchi et al., 2006; Wang and Valent, 2009). Sexual cycles can be observed on culture medium and are rarely found in the nature, but the sexual recombination can also occur in some limited regions in south Asia (Saleh et al., 2012). Moreover, hermaphroditic strains identified in some restricted areas lead to the assumption that sexual reproduction could occur in the field (Wang and Valent, 2009). Hermaphroditic isolates are rare and previous studies showed that hermaphroditic strains could be found in Thailand, India, Philippines, and Yunnan province of China (Hayashi et al., 1997; Kumar et al., 1999; Mekwatanakarn et al., 1999). In this study, hermaphroditic strains are found in four regions, three of them locate in the northeastern of the Sichuan Basin and isolates collected in five regions have both the mating types (Figure 7). These findings suggested that sexual reproduction may occur in some limited regions in the Sichuan Basin, which may generate complex blast fungus races and change the population structure through DNA exchange. Intriguingly, the most abundance of *AVR-Pib* alleles was identified in the nurseries YS, YA, and TJ (Figure 4), where they have hermaphroditic strains (Figure 7). But, two virulence *AVR-Pik* alleles, namely, *AVR-PikB and AVR-PikC*, escaped from recognition by *Pik* alleles found in the nurseries PX, NB, AZ, YA, and QW, respectively (Figure 6C), where they have single, couple, or hermaphroditic of the two mating types. It is interesting to further dissect the relationship between parasexual recombination or sexual mating and mutation, absence, or duplication of *AVR* genes. Furthermore, the distribution of either *AVR* genes or their variations has distinct geographical features (Figures 4, 6C). It may be caused by different selection pressure conferred by rice variety diversity in various regions of the Sichuan Basin. To further monitoring the dynamics of the *AVR* gene variations, we need to collect more isolates and perform further analysis in the future. Meanwhile, the pan-genome analysis on isolates from different years may help to understand the molecular mechanism of the rice blast epidemic in the Sichuan Basin.

## Data Availability Statement

The datasets presented in this study can be found in online repositories. The names of the repository/repositories and accession number(s) can be found in the article/Supplementary Material.

## Author Contributions

Y-YH and W-MW conceived the experiment. Z-JH, X-YL, HF, S-XZ, YX, X-XL, CL, R-MZ, MP, Y-PJ, X-HH, G-BL, J-HZ, HW, and D-QL carried out the experiment. W-SZ, C-HF, Z-XZ, Yu-LP, FH, and Yo-LP managed the blast nurseries. Z-JH, J-WZ, JF, YL, and MH analyzed the data. Z-JH wrote the manuscript. Y-YH and W-MW edited the manuscript. All authors contributed to the article and approved the submitted version of the manuscript.

## Funding

This study was supported by the National Natural Science Foundation of China (U19A2033 to W-MW) and the Department of Science and Technology of Sichuan Province (2020YJ0332 to W-MW and 2021YJ0304 to YL).

## Conflict of Interest

The authors declare that the research was conducted in the absence of any commercial or financial relationships that could be construed as a potential conflict of interest.

## Publisher's Note

All claims expressed in this article are solely those of the authors and do not necessarily represent those of their affiliated organizations, or those of the publisher, the editors and the reviewers. Any product that may be evaluated in this article, or claim that may be made by its manufacturer, is not guaranteed or endorsed by the publisher.
